# A Dual Role for Corneal Dendritic Cells in Herpes Simplex Keratitis: Local Suppression of Corneal Damage and Promotion of Systemic Viral Dissemination

**DOI:** 10.1371/journal.pone.0137123

**Published:** 2015-09-02

**Authors:** Kai Hu, Deshea L. Harris, Takefumi Yamaguchi, Ulrich H. von Andrian, Pedram Hamrah

**Affiliations:** 1 Schepens Eye Research Institute, Massachusetts Eye & Ear, Department of Ophthalmology, Harvard Medical School, Boston, Massachusetts, United States of America; 2 Cornea Service, Massachusetts Eye & Ear Infirmary, Department of Ophthalmology, Harvard Medical School, Boston, Massachusetts, United States of America; 3 Immune Disease Institute, Program in Cellular and Molecular Medicine at Children’s Hospital Boston, Harvard Medical School, Boston, Massachusetts, United States of America; 4 Department of Ophthalmology, Tufts Medical Center, Tufts University School of Medicine, Boston, Massachusetts, United States of America; 5 Division of Immunology, Department of Microbiology & Immunobiology, Harvard Medical School, Boston, Massachusetts, United States of America; UC Irvine Medical Center, UNITED STATES

## Abstract

The cornea is the shield to the foreign world and thus, a primary site for peripheral infections. However, transparency and vision are incompatible with inflammation and scarring that may result from infections. Thus, the cornea is required to perform a delicate balance between fighting infections and preserving vision. To date, little is known about the specific role of antigen-presenting cells in viral keratitis. In this study, utilizing an established murine model of primary acute herpes simplex virus (HSV)-1 keratitis, we demonstrate that primary HSV keratitis results in increased conventional dendritic cells (cDCs) and macrophages within 24 hours after infection. Local depletion of cDCs in CD11c-DTR mice by subconjuntival diphtheria toxin injections, led to increased viral proliferation, and influx of inflammatory cells, resulting in increased scarring and clinical keratitis. In addition, while HSV infection resulted in significant corneal nerve destruction, local depletion of cDCs resulted in a much more severe loss of corneal nerves. Further, local cDC depletion resulted in decreased corneal nerve infection, and subsequently decreased and delayed systemic viral transmission in the trigeminal ganglion and draining lymph node, resulting in decreased mortality of mice. In contrast, sham depletion or depletion of macrophages through local injection of clodronate liposomes had neither a significant impact on the cornea, nor an effect on systemic viral transmission. In conclusion, we demonstrate that corneal cDCs may play a primary role in local corneal defense during viral keratitis and preserve vision, at the cost of inducing systemic viral dissemination, leading to increased mortality.

## Introduction

Herpes simplex keratitis is the most common cause of corneal blindness in the world [1], with around 48,000 cases per year in the United States alone [2]. Corneal epithelial cells express the herpes simplex virus (HSV) entry receptors Herpes Virus Entry Mediator (HVEM), Nectin-1, Nectin-2, 3-O Sulfated Heparan Sulphate and Paired Immunoglobulin-Like type 2 Receptor-a (PILR-a) [3–4], rendering them susceptible to direct viral infection. As such, it is well-established that HSV-1 can primarily infects the cornea either directly through the "front door" (the cornea/ocular surface) [5], or indirectly through other tissues, such as the oral mucosa [6]. During primary acute herpes simplex keratitis, neuro-invasion of sensory corneal nerves takes place by the HSV-1, resulting in latency in the trigeminal ganglion (TG), and subsequent reactivation. It is the virus reactivation that leads to chronic recurrent herpes stromal keratitis [7], which is characterized with a large amount of infiltration by neutrophils, macrophages (MΦs) and T cells [8–10], and may cause severe corneal scarring. Thus, studies on primary herpes simplex keratitis are required to avoid or limit latency and subsequent recurrent herpes simplex keratitis.

Previously, significant decrease in local corneal nerves and sensation [11], as well as increase in central corneal epithelial Langerhans cells (LCs) have been demonstrated in herpes simplex keratitis [12]. Conventional dendritic cells (cDCs), which are located in the corneal epithelium and most anterior part of the stroma [13], are considered the main professional antigen-presenting cells (APCs) of the cornea [13–14], and have been implicated in T cell responses and the induction of tolerance. Recent evidence suggests that corneal APCs may play a role in the induction of latency and the severity of chronic recurrent herpetic stromal keratitis [15–16]. Further, purported early-responding corneal cDCs have recently been shown to mediate natural killer (NK) cell responses in primary HSV-1 infection [17]. Moreover, systemic depletion of MΦs, which play a critical role in systemic antiviral defense [18], resulted in increased corneal HSV titers, but had no effect on corneal scarring or latency [19]. However, the early host-pathogen interaction, the specific mechanisms of viral dissemination, and the differential roles of corneal APCs in primary acute herpes simplex keratitis remain unclear to date.

Previous studies have demonstrated evidence for bidirectional interplay between the immune and nervous system [20–22], and shown that cDCs are intimately associated with nerves [23–24]. Here we demonstrate that corneal cDCs, but not MΦs, play a role in systemic dissemination of HSV from the cornea, prevent local corneal damage, and thus preserve vision.

## Materials and Methods

### Mice

Six-to-eight week old wild-type C57BL/6 male (WT, Charles River Laboratories, Inc, Wilmington, MA), P-selectin glycoprotein ligand-1 (PSGL1) knockout (KO) [25] (C57BL/6 background, The Jackson Laboratory, Bar Harbor, Maine), and CD11c-GFP-DTR mice [26] (C57BL/6 background, The Jackson Laboratory) were bred and housed in specific pathogen free conditions. CD11c-GFP-DTR mice carry a transgene encoding a simian diphtheria toxin (DT) receptor (DTR)-green fluorescent protein (GFP) fusion protein under the control of the murine CD11c promoter, which makes them sensitive to cDC depletion with DT. Mice were randomly assigned to study groups using a Random Number Table.

### Virus

The HSV-1 strain McKrae (kindly provided by Dr. Homayon Ghiasi, Cedars-Sinai Medical Center, Los Angeles, CA), a stromal disease-causing [27], neurovirulent HSV-1 strain, and the KOS strain (kindly provided by Dr. Robert Hendricks, University of Pittsburgh, Pittsburgh, PA) were used for ocular challenge. The McKrae strain is used by several leading laboratories studying HSV keratitis. We elected to use this strain in order to study the systemic clinical effects, such as death. HSV-1 was propagated in Vero cells (derived from African green monkey kidney, kindly provided by Dr. Judy Lieberman, Children’s Hospital Boston, Boston, MA). Briefly, at maximum cytopathic effect, the virus was harvested by three cycles of freezing and thawing. After pelleting the virus at 17,000 rpm for 30 minutes, the virus was collected and stored at -80°C.

### Corneal HSV-1 infection

After mice were anesthetized by intraperitoneal (i.p.) injection of ketamine (100mg/kg) and xylazine (20 mg/kg), corneas of the right eyes were scarified 5 (horizontal) x 5 (vertical) times with a 30 gauge needles. C57BL/6 WT mice, CD11c-DTR-GFP mice or PSGL-1 KO mice corneas of the right eyes were inoculated with 2×10^5^ PFU/μl of HSV-1 strain McKrae in 10 μl of tissue culture medium (DMEM, Mediatech, Inc, Manassas, VA) topically. Administration of culture medium alone was used as sham control.

### Depletion of corneal cDCs and MΦs

CD11c-DTR-GFP mice were depleted of corneal cDCs (cDC(-)) using 30 ng of DT in 10 μl (5μl temporally and 5μl nasally) of phosphate-buffered saline (PBS) subconjunctivally (s.c.) 2 days before infection and repeated every 2 days thereafter; C57BL/6 WT mice were depleted of corneal MΦs (MΦs(-)) using 10 μl (5μl temporally and 5μl nasally) clodronate liposomes (CL; 0.05 mg/10 μl clodronate, Encapsula NanoSciences, Nashville, TN) s.c. 2 days before infection and repeated every 2 days thereafter. For concurrent depletion of cDCs and MΦs, both 30 ng DT and 0.05 mg of CL were injected into CD11c-DTR-GFP mice s.c. (cDC(-)&MΦ(-)). As a control, C57BL/6 WT mice were similarly injected s.c. with both DT and control liposomes (without clodronate, Encapsula NanoSciences, Nashville, TN) and are referred to here as sham-depleted mice. To deplete corneal cDCs and MΦs systemically, CD11c-DTR-GFP mice were given i.p. injections of 150 ng DT 2 days before HSV inoculation and every 2 days thereafter to deplete cDCs; C57BL/6 WT mice were injected with 0.25 mg CL i.p. to deplete corneal MΦs; combined DT and CL were injected for depletion of cDCs and MΦs; and both DT and control liposomes were injected to C57BL/6 WT for sham-depletion. Corneas were harvested on days 0, 3, and 6 post infection (p.i.) for immunohistochemistry staining to confirm depletion of cDCs and/or MΦs.

### Clinical evaluation of HSV keratitis

The severity of acute keratitis in mice was scored by bio-microscopy in a masked fashion on a 0–4 scale modified according to a previously described method [28]: 0, normal; 1, corneal lesions confine to less than one quarter of the diameter of the cornea and iris is visible; 2, corneal lesions are between one quarter and one half of the diameter of the cornea and iris is visible; 3, corneal lesions extend over more than one half of the diameter of the cornea and iris is partially invisible; and 4, corneal lesions spread over the whole cornea and iris is completely invisible. The representative clinical corneal photographs were taken through a bio-microscope.

### Monitoring mice survival rate

CD11c-DTR-GFP or C57BL/6 WT mice were depleted of cDCs or MΦs or both, or sham-depleted as stated above (4 groups, n = 20/group). Mice were monitored every 12 hours for survival until 20 days p.i. Mice were given buprenorphine (0.1 mg/kg subcutaneously) to minimize animal suffering if any pain/distress (ocular swelling; red and discharge; inactivity; lack of food or water intake; changes in gait) was noted; if suffering severe pain/distress (ruffled fur; hunched posture; crouching; shivering), those mice were euthanized by CO_2_ inhalation followed by cervical dislocation, and were counted as endpoints (didn’t survive). We did not find any unexpected deaths during the process.

### Measurement of virus titers in corneas

On days 1, 3, 5, and 7 p.i., mice were sacrificed by CO_2_, and corneas were harvested with Vannas scissors in a sterile fashion. HSV-1 viral titers within tissues were determined by homogenizing the corneal tissues in DMEM (Mediatech, Inc, Manassas, VA) with gentleMACS™ Dissociator (Miltenyi Biotec, Bergisch Gladbach, Germany), clarifying the supernatant (10,000 × g, 5 min, Allegra® X-15R, Beckman Coulter Inc, Brea, CA), and assaying the supernatant by typical virus plaque assay on Vero cells. Briefly, 100μl volumes containing serial dilutions of the supernatant were plated on Vero cell monolayers in 6 well plates and incubated at 37°C for 1 h. Monolayers were rinsed, overlaid with 0.5% methylcellulose (Sigma-Aldrich, Germany) in DMEM/5% calf serum, and incubated at 37°C for 2–3 days. Plates were then stained with 1% crystal violet (Sigma-Aldrich, Germany) and plaques were counted.

### Detection of HSV-1 mRNA by qRT-PCR

Ipsilateral and contralateral TGs, submandibular lymph nodes (corneal draining lymph nodes [dLNs]), and corneas, were excised at different time points. For detecting the levels of HSV-1 invading the corneal stroma, the stroma was separated from the epithelium as previously described [29]. Briefly, freshly excised corneas were immersed in PBS, containing 20 mM EDTA (VWR, West Chester, PA) at 37°C for 1 hour. The epithelium was removed from the underlying stroma with forceps and the stromal layer washed in PBS. Corneal stromas, TGs or dLNs were immersed in RNAlater (Ambion, Foster City, CA) RNA stabilization reagent, and stored at -20°C until processing. Total tissue RNA was extracted and purified with RNeasy Tissue Mini Kit (QIAGEN, Germantown, MD) as described by the manufacturer. RNA yield was measured by spectroscopy (NanoDrop ND-1000; NanoDrop Technologies, Inc., Wilmington, DE). The RNA was then retrotranscribed to cDNA with random hexamer primers by using iScript cDNA Synthesis Kit (Bio-Rad, Hercules, CA), in accordance with the manufacturer’s instructions. The expression levels of the HSV-1 glycoprotein B (gB) gene, along with the endogenous control GAPDH (glyceraldehyde-3-phosphate dehydrogenase) gene [30], were evaluated using commercial SYBR Premix EX.Tag kit (Takara, Japan). The following primers were used for gB: 5’-AACGCGACGCACATCAAG-3’(forward) and 5’-CTGGTACGCGA TCAGAAAGC-3’(reverse). Quantitative real-time PCR (qRT-PCR) was performed using the qPCR machine (Bio-Rad iCycler, Hercules, CA) in 96-well plates, and all reactions were performed in a final volume of 20 μl. Briefly, all mixtures contained 2 μl of cDNA template (around 5 ng), 10 μl SYBR mix, 2 μl primers and 6 μl dd H_2_O. Universal thermal cycling conditions were as follows: after an initial 30s at 95°C, the samples were cycled 40 times at 95°C for 10s, 55°C for 10s and 72°C for 30s. Relative gene expression levels were normalized to the expression of the GAPDH housekeeping gene (endogenous control) and calculated using the comparative threshold cycle (*C*_*T*_).

### Immunofluorescence immunohistochemistry and confocal microscopy

Corneas were excised from above-mentioned four experimental groups, and fixed in 100% chilled acetone for 15 minutes at room temperature. Corneas were then incubated in 2% bovine serum albumin (BSA) and 1% anti-FcR monoclonal antibody (CD16/CD32) diluted in PBS for 45 minutes to block non-specific staining, and immunostained with multiple fluorescence-conjugated antibodies overnight at 4°C. Previous studies have demonstrated that staining of corneal whole-mounts results in penetration of the whole cornea [14, 31]. Antibodies used were FITC-conjugated rabbit-anti-HSV-1(Dako,Carpinteria,CA,http://www.dako.com/us/ar38/p235587/prod_products.htm); NL557-conjungated mouse anti-βIII-Tubulin (R&D, Minneapolis, MN, http://www.rndsystems.com/Products/NL1195R); PE-conjugated Armenian Hamster anti-mouse CD11c, Alexa fluor-647-conjugated rat anti-mouse F4/80 and Alexa fluor-647-conjugated rat anti-mouse CD45 (all Biolegend, San Diego, CA, http://www.biolegend.com/); FITC-conjugated rat anti-mouse MHC-II (BD, San Jose, CA,http://www.bdbiosciences.com/external_files/pm/doc/tds/mouse/live/web_enabled/06344D_553623.pdf); Alexa fluor 488-conjugated rabbit anti-GFP (Invitrogen, Grand Island, NY, http://products.invitrogen.com/ivgn/product/A21311). Isotype controls used were FITC-conjugated rabbit IgG (Dako); NL557-conjungated mouse IgG2a isotype control (R&D); PE Armenian Hamster IgG, Alexa fluor-647-conjugated rat IgG2a,κ, and Alexa fluor-647-conjugated Rat IgG2b,κ isotype controls (all Biolegend); FITC-conjugated Rat IgG2a,κ (BD); rabbit IgG isotype control (Invitrogen). Three thorough washings in PBS followed every step for 5 minutes each. Finally, corneas were covered with a mounting medium (Vector laboratories, Inc, Burlingame, CA) and analyzed by a confocal microscope (FV1000, Olympus, Japan). Central and peripheral areas for each cornea were assessed separately, with the central area defined as the area within 0.6 mm of the corneal center, and the periphery defined as being within a 0.9-to 1.5-mm radial distance from the center. Three different fields of each central or peripheral cornea were examined respectively. At least three different corneas were examined per each experiment. Multiple z-sections were generated throughout the cornea, and 3D movie were generated by Imaris software (Bitplane Inc, South Windsor, CT) as necessary.

### Image quantification and analysis

Cell numbers for each image were counted in a masked fashion with Image J software (NIH, Bethesda, MD) manual model and the density was attained divided by image frame area. Nerve length of each image was measured in a masked fashion with Neuron J software (NIH, Bethesda, MD) and the density was attained divided by image frame area. For quantification of percentage of infected nerves, the infected nerve length and the total nerve length were measured separately in the same image frame as described above, and the percentages were calculated. Three different fields of each central or peripheral cornea were counted respectively and averaged. Each image counting or measuring repeated twice and averaged.

### Statistical analysis

Statistical analysis was performed with the SPSS software 16.0 (SPSS Inc. Chicago, IL). Results were expressed as mean ± SD. The data is the representative from three repeated experiments. One-Way ANOVA was used for comparisons of means among three or more groups with Bonferroni’s post-hoc test (the normal distribution and homogeneity of variance of the data were confirmed before comparisons); Student’s t-test was used for comparison between two groups (the normal distribution of the data was confirmed before comparison); Chi-square test was used for comparison of rates between two groups; Fisher Exact Probability Test was used for comparison of rates between two groups with small numbers per group. Results were considered statistically significant when the *P* value was <0.05.

### Ethics statement

Mouse experiments were approved at two locations: Harvard Medical Area (HMA) Standing Committee on Animals Protocol #04612: Immunobiology Of Corneal Antigen Presenting Cells and Schepens Eye Research Institute Institutional Animal Care and Use Committee Protocol #S-344-0415: To study the Trafficking Mechanisms and Function of Corneal Antigen Presenting Cells in Health, Immune and Inflammatory Diseases. At both, all experiments were performed in accordance with the guidelines for Care and Use of Laboratory Animals of the National Institutes of Health and all animals were treated according to the ARVO Statement for the Use of Animals in Ophthalmic and Vision Research.

## Results

### Corneal HSV-1 inoculation results in rapid maturation and differential increase of antigen-presenting cells

**Fig 1:**
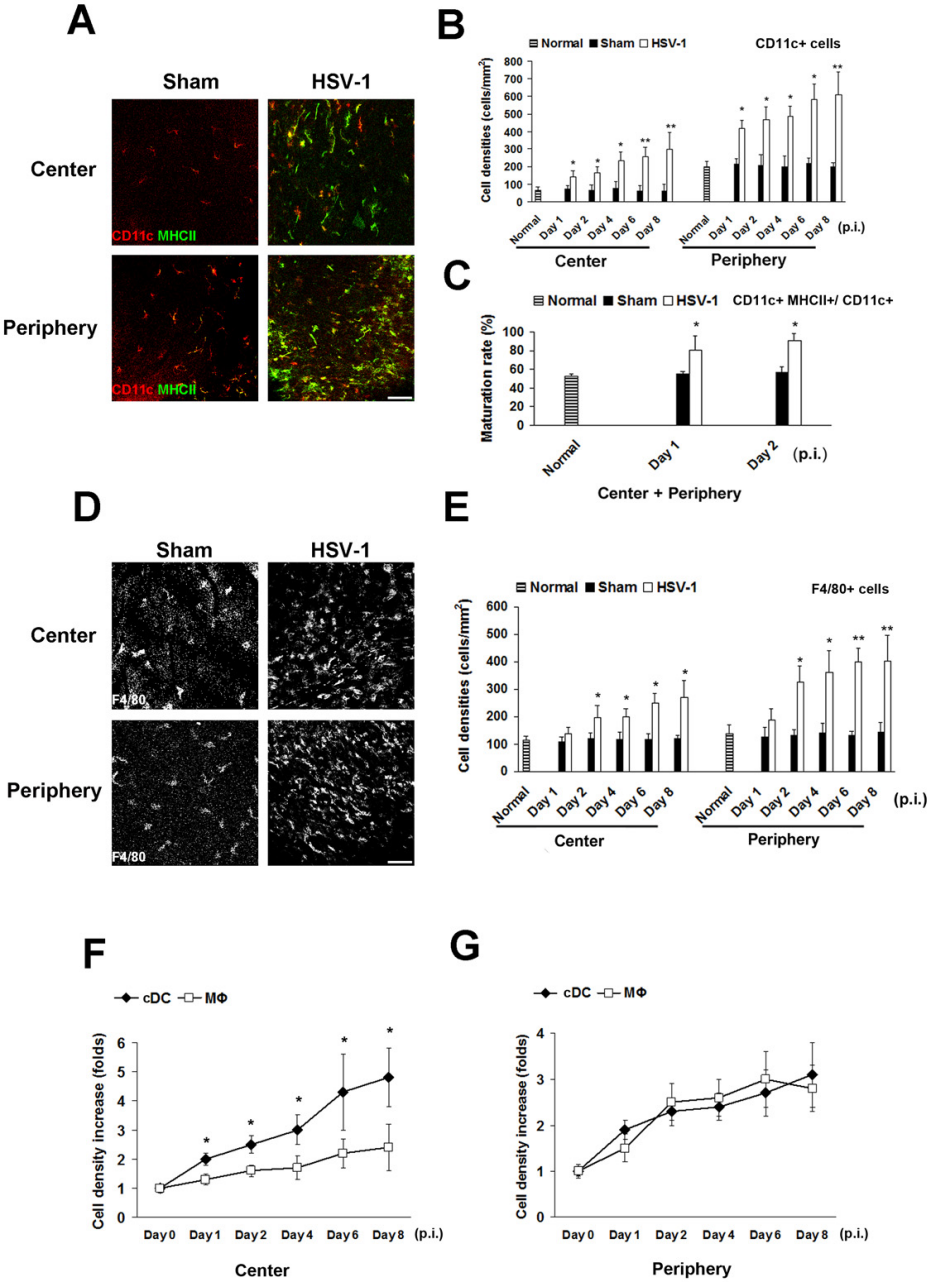
Herpes simplex keratitis results in significant increased conventional dendritic cell and macrophage density, as well as increased maturation of dendritic cells. Corneal inoculation was performed with HSV-1, or virus culture medium as sham controls, on days 1, 2, 4, 6, 8 p.i., Corneas (n = 5/time point) were excised and stained with antibodies against CD11c, F4/80, MHC-II; normal corneas were used as controls. **A:** Representative micrographs of CD11c (conventional dendritic cells [cDCs]) and MHC-II (maturation marker) expressing cells in the HSV-1 infected corneas compared with sham-infected corneas. **B:** As early as day 1 p.i., cDC densities of central and peripheral corneas were significantly increased as compared to sham controls, and continuously increased on days 2, 4, 6, 8 p.i. **C:** cDC maturation (CD11c+MHC-II+/total CD11c+) after HSV-1 infection significantly increased vs. sham controls. **D:** Representative micrographs of F4/80 expressing cells (macrophages [MΦs]) in HSV-1 infected corneas compared with sham controls. **E:** MΦ density were significantly increased in central and peripheral corneas after HSV-1 infection as compared to sham controls on days 2, 4, 6, 8 p.i. **F, G:** The relative increase in density (density after HSV infection /density after sham infection) of cDC was significant higher than the relative increase in MΦ density in central corneas on days 1, 2, 4, 6, 8 p.i., while no significant differences were found in peripheral corneas. Each experiment was replicated three times. *p<0.05, **p<0.01 compared to sham controls (Student’s t-test, two-sided); data are shown as mean ± SD. Scale bar: 100 μm.

We previously described resident populations of central corneal APCs (including cDCs and MΦs), which are immature during steady state [13–14, 29]. To understand the dynamics of APCs in primary herpes simplex keratitis, corneas were inoculated with the virulent McKrae strain. Corneas underwent immunohistochemistry for the cDC marker CD11c, the MΦ marker F4/80, and the maturation marker MHC-class II daily for 8 days. We chose a single marker for macrophages as F4/80 is a comparatively specific marker for MΦs [32–37] and F4/80-negative MΦs have, to our knowledge, not been described in the cornea [32–33]. The results demonstrated an immediate and significant increase of cDCs in central and peripheral cornea (Fig 1A and 1B) on day 1 post inoculation (p.i.)(142±33 and 415±46 cells/mm^2^) as compared to normal (65±21 and 202±28) and sham controls (72±19 and 215±29), which continuously increased to day 8. Importantly, cDC maturation increased significantly within 24 hours to 81±15%, as compared to normal (53±3%) and sham (55±3%, *p* = 0.02; Fig 1C). Similarly, MΦ density increased p.i. (Fig 1D and 1E), although no change in MHC-II expression was observed (data not shown). However, MΦ increase was slower and less pronounced in the central cornea as compared to increase in cDCs (Fig 1F and 1G). These results suggest that corneal cDCs undergo maturation and respond immediately to viral exposure.

### Local corneal antigen-presenting cell depletion is successful and more efficient than standard systemic depletion

**Fig 2:**
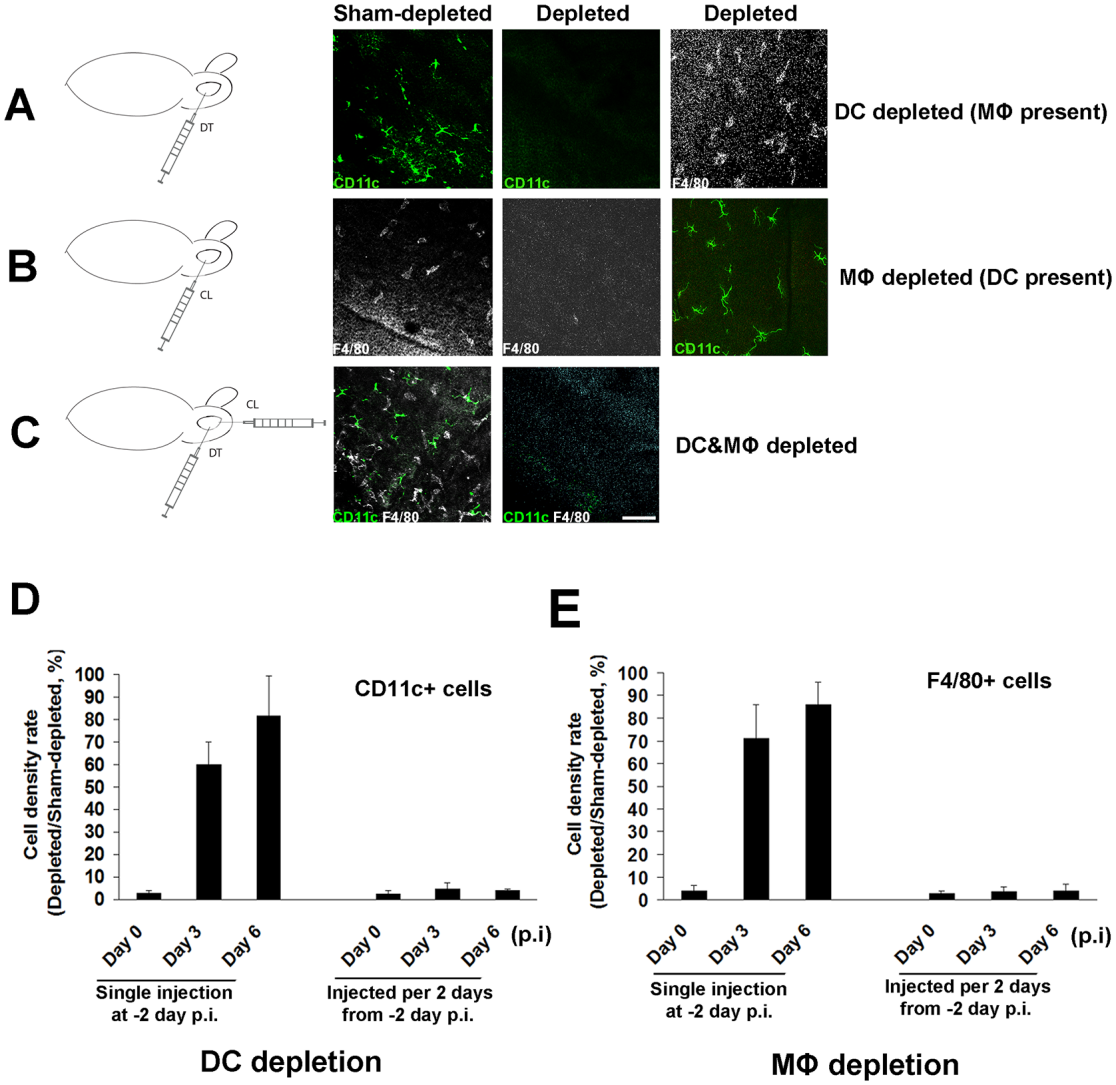
Corneas can be successfully single- or double-depleted of conventional dendritic cells and macrophages. Two days prior to HSV-1 infection (single injection), or every 2 days (multiple injections, started on -2 day p.i.), diphtheria toxin (DT), clodronate liposome (CL) or both, were injected subconjunctivally (s.c.) into CD11c-DTR transgenic or C57BL/6 WT mice in order to deplete corneal cDCs, MΦs or both; C57BL/6 WT mice injected with the mixture of DT and control liposomes (without clodronate) were used as sham-depleted controls. On days 0, 3, 6 p.i., corneas (n = 5/time point) were collected for immunostaining. **A:** Representative micrographs showing that CD11c+ cells (cDCs) were depleted, but the F4/80+ cells (MΦs) were present after s.c. DT injection. **B:** MΦs were depleted, but cDCs were present after s.c. CL injection. **C:** cDCs and MΦs were double-depleted after s.c. injection of DT and CL. **D, E:** After a single injection of DT on day -2 p.i., cDCs were almost completely depleted on day 0 p.i. However, on day 3 p.i., corneas repopulated with cDCs to 60% of normal corneal levels, and increased to more than 80% on day 6 p.i.; with repeated injections of DT every 2 days, cDCs remained depleted by more than 95% on days 0, 3, 6 p.i. (D). MΦs (E) or cDCs & MΦs (S1C Fig) were similarly depleted with repeated s.c. injections of CL or DT and CL every 2 days. Each experiment was replicated three times. Data are shown as mean ± SD. Scale bar: 100 μm.

To study the functional relevance of corneal cDCs and MΦs, we next aimed to develop modified models to deplete them in the cornea specifically or in combination. Initial intraperitoneal (i.p.) injection of Diphtheria Toxin (DT) in CD11c-GFP-DTR mice to deplete corneal cDCs, and clodronate liposomes (CL) to deplete MΦs, resulted in only 43.4±8.0% and 36.1±12.1% depletion of total corneal cDCs and MΦs respectively (S1A and S1B Fig). Repeated i.p. DT or CL injections did not lower cDC or MΦ density. These results were not surprising in light of the fact that the cornea is an avascular tissue. However, cDCs, but not MΦs, were effectively depleted (97.2±2.1% at day 0) with a single subconjunctival (s.c.) injection at -2 days p.i. (Fig 2A and 2D; S1A Fig). Similarly, s.c. injection of CL (Fig 2B and 2E; S1B Fig), resulted in 96.0±2.9% MΦs, but not cDCs, depletion. Despite cDCs (59.9±10.2%) and MΦs repopulation within 3 days, repeated s.c. DT or CL injections every 2 days resulted in continuous cDC- and MΦ-depletion (Fig 2D and 2E; S1A and S1B Fig). Moreover, concurrent s.c. injections of both DT and CL to CD11c-GFP-DTR mice every 2 days resulted in their effective continuous depletion (Fig 2C and S1C Fig). This model can deplete corneal cDCs and/or MΦs of both infected (Day 3, 6 of right part of Fig 2D and 2E and S1C Fig) and non-infected (Day 0 of Fig 2D and 2E, S1C Fig) mice. Further, s.c. administration comparatively depletes local corneal cells specifically, and does not deplete cells in other organs, such as draining lymph node (S1D–S1F Fig). Thus, these modified local models of cDC and MΦ depletion allow us to study their roles in corneal infectious and immune-mediated diseases.

### Depletion of local corneal dendritic cells, but not macrophages, results in increased severity of primary herpetic keratitis and local corneal damage

**Fig 3:**
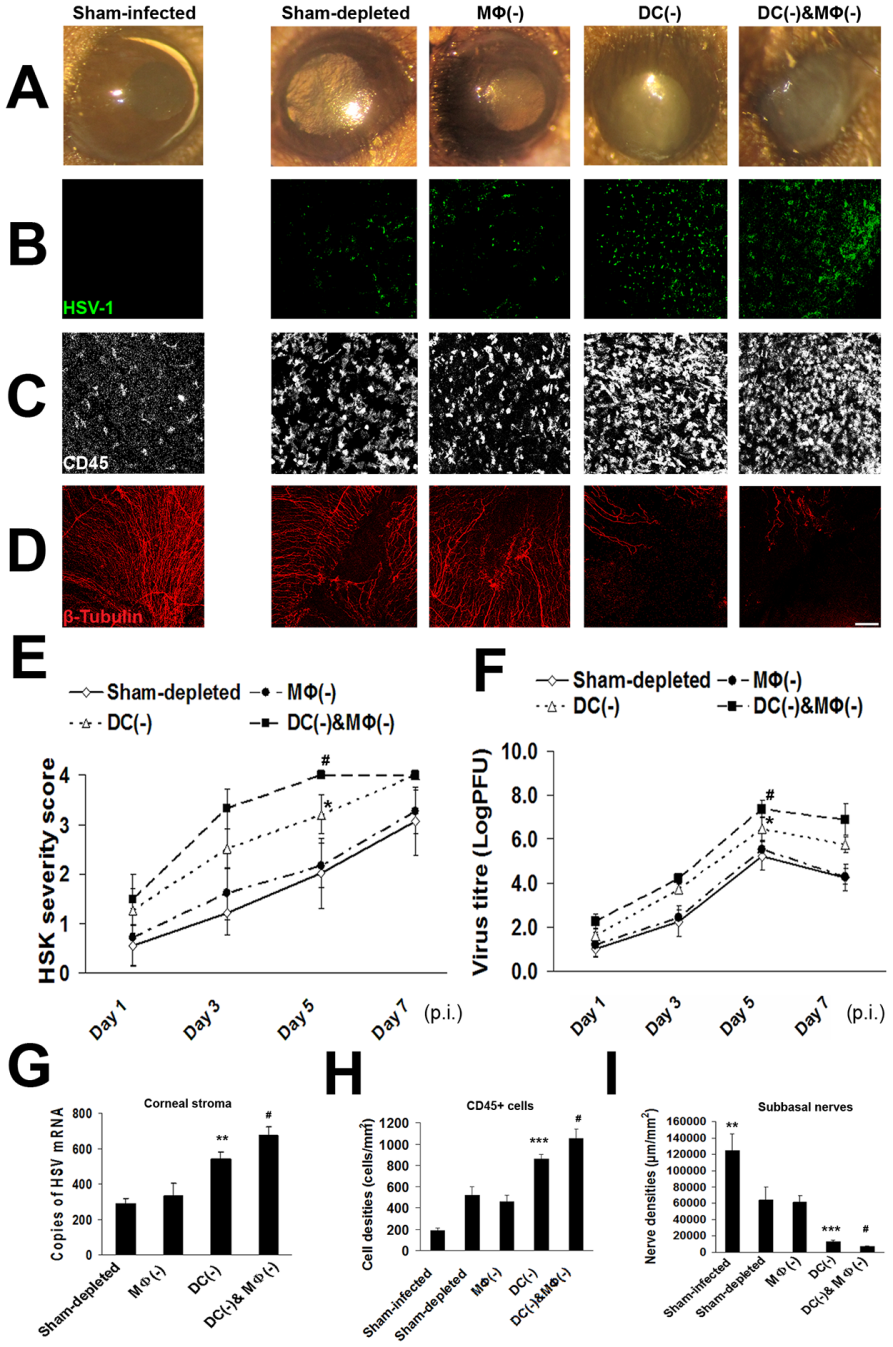
Upon depletion of corneal conventional dendritic cells, virus titers and the local corneal damages are significantly increased in primary herpes simplex keratitis. Infected corneas (n = 5/time point) were collected and virus titers, HSV mRNA levels measurement, or immunohistochemistry studies were performed. cDC and MΦ depletion were compared to sham-depleted infected mice after subconjunctival injections of DT and CL into CD11c-GFP-DTR or C57BL/6 WT mice, as well as to sham-infected mice without active viral inoculation (culture medium alone). Keratitis severity was graded (n = 5/time point). **A:** Representative corneal photos of primary acute herpes simplex keratitis severity of the mice in different groups are shown. **B:** Representative micrographs of viral invasion into the corneal stroma of the mice in different groups are shown. **C:** Representative micrographs of inflammatory cells (CD45^+^) infiltrating into the corneas of the mice in different groups are shown. **D:** Representative micrographs of nerve destructions of the mice in different groups are shown. **E:** The keratitis severity after HSV inoculation in cDC depleted (cDC(-)) mice was significantly increased as compared sham-depleted and MΦ-depleted (MΦ(-)) mice. The keratitis severity with cDC/MΦ double-depletion (cDC(-)&MΦ(-)) was further increased compared to cDC(-) mice. **F, G:** The viral titer levels (day 5 p.i.) of corneas and HSV-1 mRNA of the corneal stroma were significantly increased in cDC(-) corneas compared to sham controls or MΦ depletion. Double-depletion of cDCs and MΦs showed significantly HSV levels compared to cDC depletion alone. **H:** Increased inflammatory (CD45+) cells infiltrated corneas with cDC depletion (day 5 p.i.) compared to controls. cDC and MΦ double depletion resulted in increased CD45+ cells compared to cDC depletion alone. **I:** Corneal nerve density was significantly decreased in cDC depleted or cDC and MΦ double-depleted corneas as compared to sham controls or MΦ depleted corneas. Each experiment was replicated three times. *p<0.05, **p<0.01 and *** *p*<0.001 compared with sham-depleted; ^**#**^ *p*<0.05 compared with cDC depletion (ANOVA with Bonferroni’s post-hoc test, two-sided). Data are shown as mean ± SD. Scale bar: 100 μm.

To study the functional role of APCs, we applied our local depletion models. Corneal HSV inoculation in sham-depleted mice resulted in keratitis with epithelial defects and corneal opacification from day 1 p.i. (0.6±0.5), further increasing to day 5 (2.0±0.7) (Fig 3A and 3E). While MΦ-depletion did not alter the clinical course (Fig 3A and 3E), continuous cDC-depletion resulted in increased primary herpes simplex keratitis severity as early as day 1 p.i., reaching significance on day 5 (*p* = 0.03). Moreover, continuous cDC/MΦ double-depletion resulted in earlier and more severe keratitis (4.0), vs. cDC-depletion alone (3.2±0.4) (Fig 3A and 3E).

In order to determine if the increased cDC depletion-induced keratitis is due to increased viral levels, corneas were stained with anti-HSV-1 antibody, which reacts with all major glycoproteins present in the viral envelope, to determine the depth of stromal viral invasion (Fig 3B). In addition, HSV titers (Fig 3F) and HSV mRNA levels (Fig 3G) were measured for all groups in homogenized corneas. Compared to HSV-inoculated sham- and MΦ-depleted groups, cDC-depletion (and cDC/MΦ double-depletion) resulted in increased stromal viral invasion. Similarly, cDC-depletion resulted in higher viral titers at all time points (*p* = 0.04 for day 5 p.i.; Fig 3F). Moreover, cDC-, but not MΦ-depletion, resulted in significantly increased stromal viral mRNA levels on day 5 p.i. (peak of viral titers), vs. sham depletion (*p* = 0.004; Fig 3G), with mRNA levels being even higher with cDC/MΦ double-depletion.

Next, to study the role of cDCs and MΦs on the corneal inflammatory response, corneas were stained for the pan-leukocyte marker CD45 to assess leukocyte infiltration. HSV-infected, sham-depleted corneas demonstrated significant increase in CD45^+^ cells (360±55 cells/mm^2^) as early as day 1 p.i. vs. sham-infected mice (171±15, *p* = 0.005), and plateaued at day 5 (Fig 3C and S2 Fig). Depletion of cDCs, but not MΦs, resulted in significantly increased leukocyte infiltration at day 1 p.i (650±60, *p* = 0.01), and increasing to day 5 (860±50), with cDC/MΦ double-depletion resulting in additional increased leukocyte infiltration (*p* = 0.03).

We have recently shown that patients with herpes simplex keratitis demonstrate a significant loss of subbasal corneal nerves within the first week of infection [11]. To understand the mechanism and dynamics of nerve destruction and the potential role of APCs in this process, HSV-infected corneas were stained for the neuronal marker βIII-Tubulin, and corneal nerve density was assessed (Fig 3D and 3I; S3 Fig). As early as day 1 p.i., profound nerve loss was observed in HSV-infected, sham-depleted corneas (64,200±15,390 μm/mm^2^) vs. sham-infected eyes (124,500±20,460; *p* = 0.003), with dramatic progressive nerve loss until day 3 (1,340±520; S3A Fig). While similar levels were observed with MΦ-depletion vs. sham-depletion, cDC-depletion resulted in more severe and almost complete nerve loss than sham-depletion on day 1 p.i. (13,210±1,560, *p* = 0.0004), and cDC/MΦ double depletion resulting in even more severe nerve loss. Corneal nerve loss is not strain specific, and not related to scarification as both corneal infection with McKrae virus without corneal scarification or infection with the milder KOS virus resulted in the nerve damage as well (S3B–S3E Fig).

Collectively, our data demonstrate a key role of corneal cDCs in limiting the clinical keratitis severity, viral replication, leukocyte infiltration, and corneal nerve loss.

### Corneal nerve loss in primary herpes simplex keratitis is due to viral replication and not leukocyte infiltration

**Fig 4:**
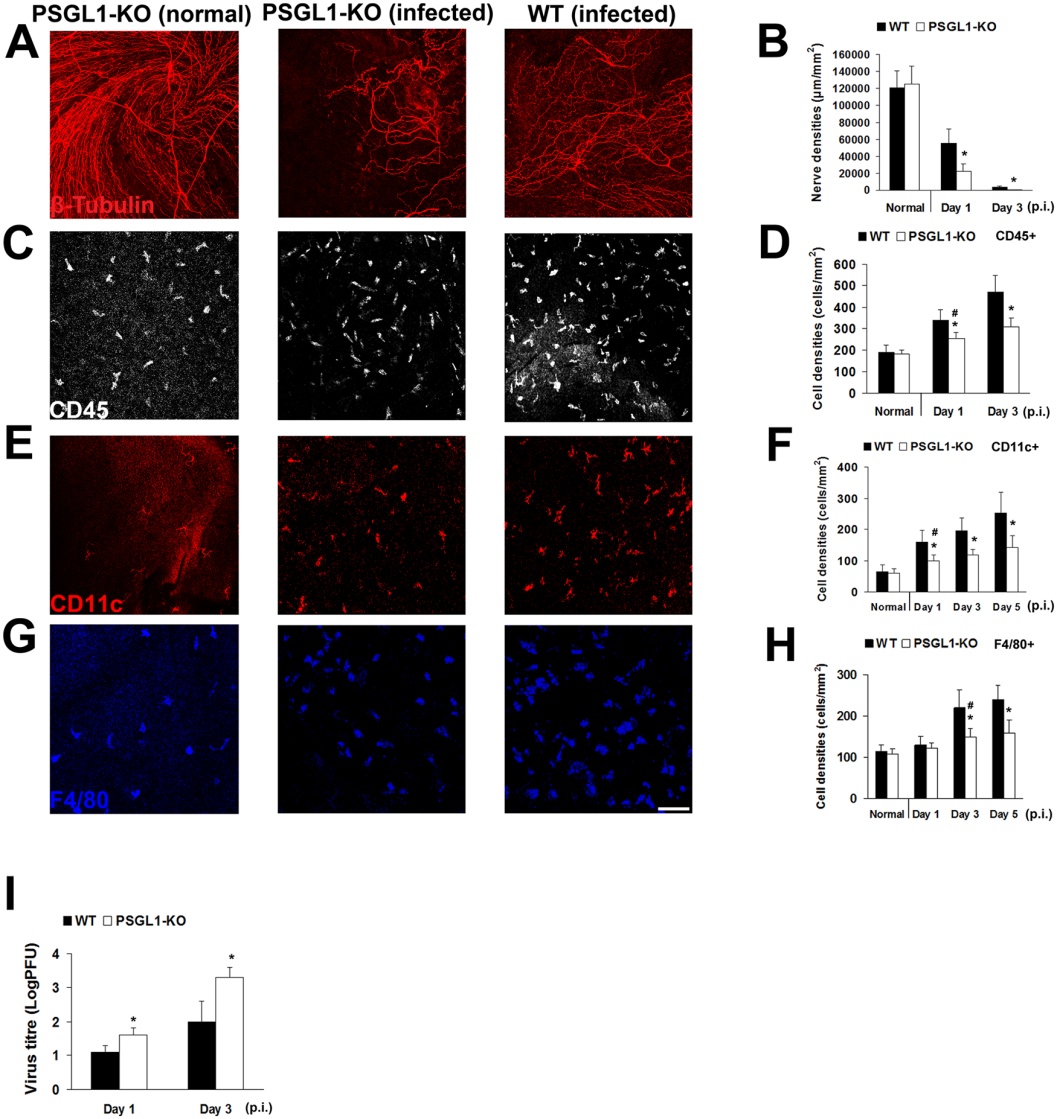
Corneal nerve damage in primary acute herpes simplex keratitis is due to viral proliferation and not corneal inflammation. **A, B:** Corneal nerve density was significantly decreased in PSGL1-KO mice as compared to WT mice after HSV-1 infection (n = 5/time point). **C, D:** The number of corneal inflammatory cells was significantly decreased in PSGL1-KO mice compared to WT mice after HSV infection (n = 5/time points). **E, F:** Corneal cDC density was significantly decreased in PSGL1-KO mice compared to WT mice after HSV infection. The increase of cDCs in PSGL-1 KO mice as compared to normal controls suggests potential differentiation from progenitor cells or proliferation (n = 5/time point). **G, H:** The number of corneal MΦs was significantly decreased in PSGL1-KO mice as compared to WT mice after HSV infection (n = 5/time points). **I**: Corneal viral titers were significantly increased in PSGL1-KO mice compared with WT mice after HSV-1 infection (n = 5/time point). Each experiment was replicated three times. *p<0.05 compared with WT; ^**#**^ *p*<0.05 compared with normal (Student’s t-test, two-sided). Data are shown as mean ± SD. Scale bar: 100 μm.

To determine if nerve loss in herpes simplex keratitis is a result of leukocyte infiltration (inflammation) vs. viral replication, we made use of P-selectin glycoprotein ligand-1 knock-out (PSGL-1 KO) mice (Fig 4). Lack of PSGL-1, which is expressed on leukocytes and required for adhesion to the vascular endothelium, will limit their infiltration into peripheral tissues. As expected, HSV-inoculation in PSGL-1KO mice resulted in significant diminishment of corneal leukocytes vs. WT mice on day 1 (255±27 vs. 340±49 cells/mm^2^, *p* = 0.03) and day 3 (310±39 vs. 470±77, *p* = 0.02). Similarly, while cDC and MΦ increase seen in WT mice in primary acute herpes simplex keratitis was limited in KO mice, their increase suggested proliferation or differentiation from local progenitor cells [14]. Further, a more profound nerve loss was observed in PSGL-1 KO vs. WT mice on days 1 (22,060±9,090 vs. 55,400±16,980, *p* = 0.02) and 3 p.i. (560±148 vs. 3,869±986, *p* = 0.01, Fig 4A), suggesting that leukocyte infiltration did not result in nerve destruction. Increased viral titers observed in PSGL-1 KO mice vs. WT mice on days 1 and 3 p.i. (Fig 4I), further suggest their role in acute corneal nerve loss.

### Corneal dendritic cells mediate transmission of herpes simplex virus to peripheral corneal nerves and induce peripherilization of the virus from the cornea to systemic compartments

**Fig 5:**
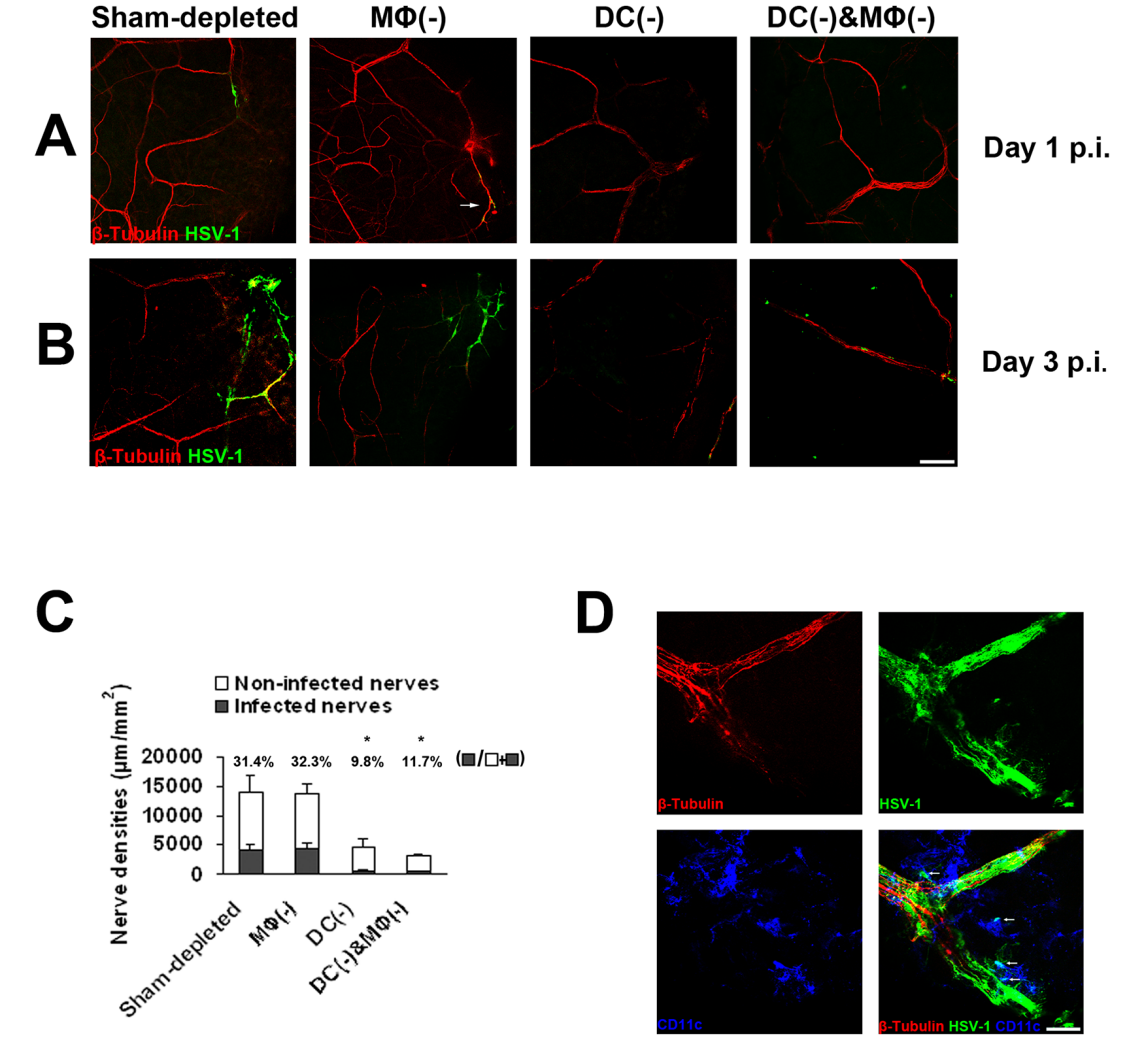
Corneal nerves demonstrate delayed and decreased HSV-1 infection in conventional dendritic cell-depleted corneas. After corneal depletion of cDCs or/and MΦs, or sham depletion, infected corneas (n = 5) were collected for immunohistochemistry studies. **A:** As early as day as 1 p.i., peripheral corneal nerves co-stained with HSV-1 in sham-depleted or MΦ depleted corneas, while depletion of corneal cDC resulted in no corneal nerve staining with HSV-1. **B, C:** On day 3 p.i., the percentage of infected nerves was significantly lower with cDC depletion as compared to sham controls and MΦ depleted corneas. **D:** The representative micrographs show that infected cDCs (arrows) were located adjacently to the infected nerves. Each experiment was replicated three times. *p<0.05 compared with sham-depleted (chi-square test). Data are shown as mean ± SD. Scale bar: 100 μm (A, B); 50μm (D).

To study if increased keratitis severity and viral titers after cDC-depletion could be due to limiting viral access to peripheral nerves, corneas were double-stained for HSV and β III-tubulin. Corneal cDCs could be located in close proximity to corneal nerves (S3F Fig). No co-staining of HSV-1 with central corneal nerves was found at any time point. However, while 31.4% of peripheral corneal nerves co-stained with HSV-1 as early as day 1 p.i. (Fig 5A–5C), cDC depletion resulted in only 9.8% (*p* = 0.01) co-staining on day 3, suggesting limited corneal nerve infection. Interestingly, while cDC/MΦ double-depletion resulted in more severe keratitis, this did not translate in increased nerve infection, suggesting that MΦ do not play a role in HSV transmission to corneal nerves. Of note, HSV-1 infected cDCs (S1 Movie), but no infected MΦs, were found in close proximity to infected corneal nerves (Fig 5D).

**Fig 6:**
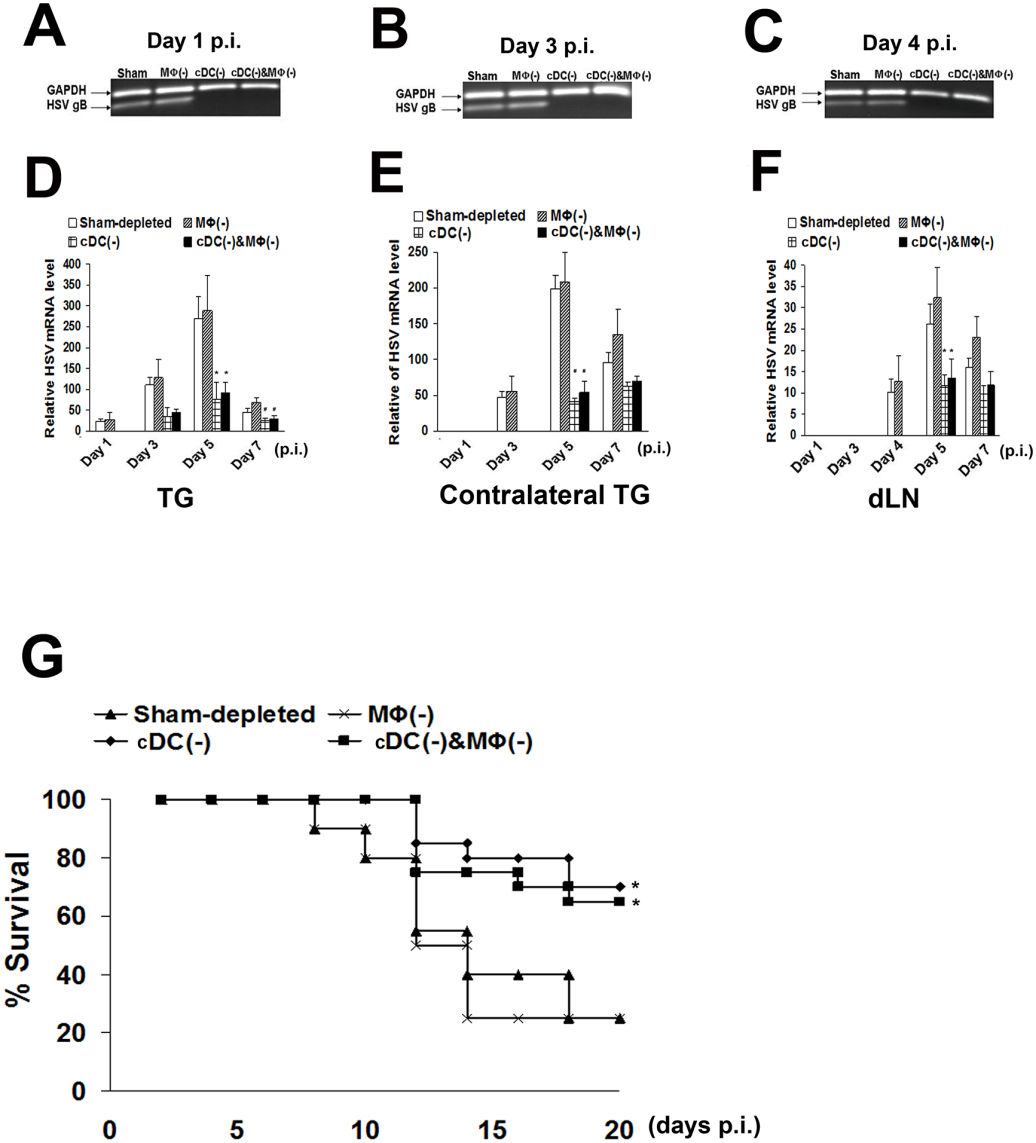
Corneal conventional dendritic cell depletion results in delayed and decreased systemic viral transmission and subsequent increase in survival rate after corneal HSV-1 infection. After corneal HSV-1 infection, trigeminal ganglions (TGs) or draining lymph nodes (dLNs) (n = 6/time point) were collected and HSV-1 mRNA levels were measured. The survival rates of infected mice were monitored (n = 20). **A, D:** On day 1 p.i, HSV mRNA was detected in the TGs of sham-depleted or MΦ-depleted mice, but not in cDC-depleted (single or double-depleted with MΦ) mice. Viral mRNA was detected on day 3 p.i. in the TGs of mice with corneal cDC-depletion, but was significantly lower as compared to sham-depleted and MΦ-depleted mice. **B, E:** On day 4 p.i, viral mRNA was detected in dLNs of sham-depleted or MΦ-depleted mice, but not in mice with corneal cDC-depletion. However, on day 5 p.i., viral mRNA was detected in dLNs of mice with corneal cDC-depletion, but at significantly lower levels than in sham-depleted controls. **C, F:** HSV mRNA was also detected in the contralateral TGs with 2 days delay, and corneal cDC-depletion resulted in delayed and decreased virus levels. **G:** Survival rates of mice with corneal cDC-depletion or mice with corneal cDC/MΦ double-depletion were significantly increased as compared to sham-depleted mice, or MΦ-depleted mice. Each experiment, including the survival experiments, was replicated three times. *p<0.05, ^**#**^ *p*<0.01 compared with sham-depleted corneas (ANOVA-D, E, F, two-sided; Fisher Exact Probability Test-G). Data are shown as mean ± SD.

To determine if differential findings of infected corneal nerves resulted in altered systemic viral transmission, draining lymph node (dLN), as well as ipsilateral and contralateral TG of mice underwent quantification for HSV gB mRNA. While HSV mRNA was detected as early as day 1 p.i in sham- and MΦ-depleted TG, peaked on day 5, and decreased on day 7, cDC-depletion resulted in significantly delayed and diminished expression in the ipsilateral TG (Fig 6A and 6D). Similarly, while HSV mRNA was detected in the dLNs of infected mice on day 4 p.i, peaked on day 5, and decreased on day 7, cDC-, but not MΦ-depletion, resulted in a significant delay and decreased expression in dLNs (Fig 6C and 6F). Surprisingly, HSV mRNA was also detected in the contralateral TG on day 3 p.i. (Fig 6B and 6E), and was diminished with ipsilateral DC depletion. We did not detect HSV mRNA in the contralateral corneas (S4A and S4B Fig), suggesting that HSV in the contralateral TG (S4C Fig) was likely transmitted through the central nervous system (CNS), and not through peripheral contamination of corneas.

### Corneal dendritic cell depletion results in increased survival through limited systemic viral transmission

The murine model of primary herpes simplex keratitis can result in death, through viral transmission to the CNS [38]. To study if limited systemic HSV transmission to the TG after cDC-depletion results in increased survival, HSV-inoculated mice were followed for 20 days. Given the high virulence of the McKrae HSV strain, only 25% of sham-depleted (5/20) and MΦ–depleted (5/20) mice survived at 20 days, with death beginning at 8 days post infection. Surprisingly, cDC-depletion resulted in significant increase in survival of 70% of mice (14/20, *p* = 0.01). The McKrae HSV strain was particularly chosen to study the effect on survival as well, as milder strains, such as the KOS strain do not affect survival rate of mice.

**Fig 7:**
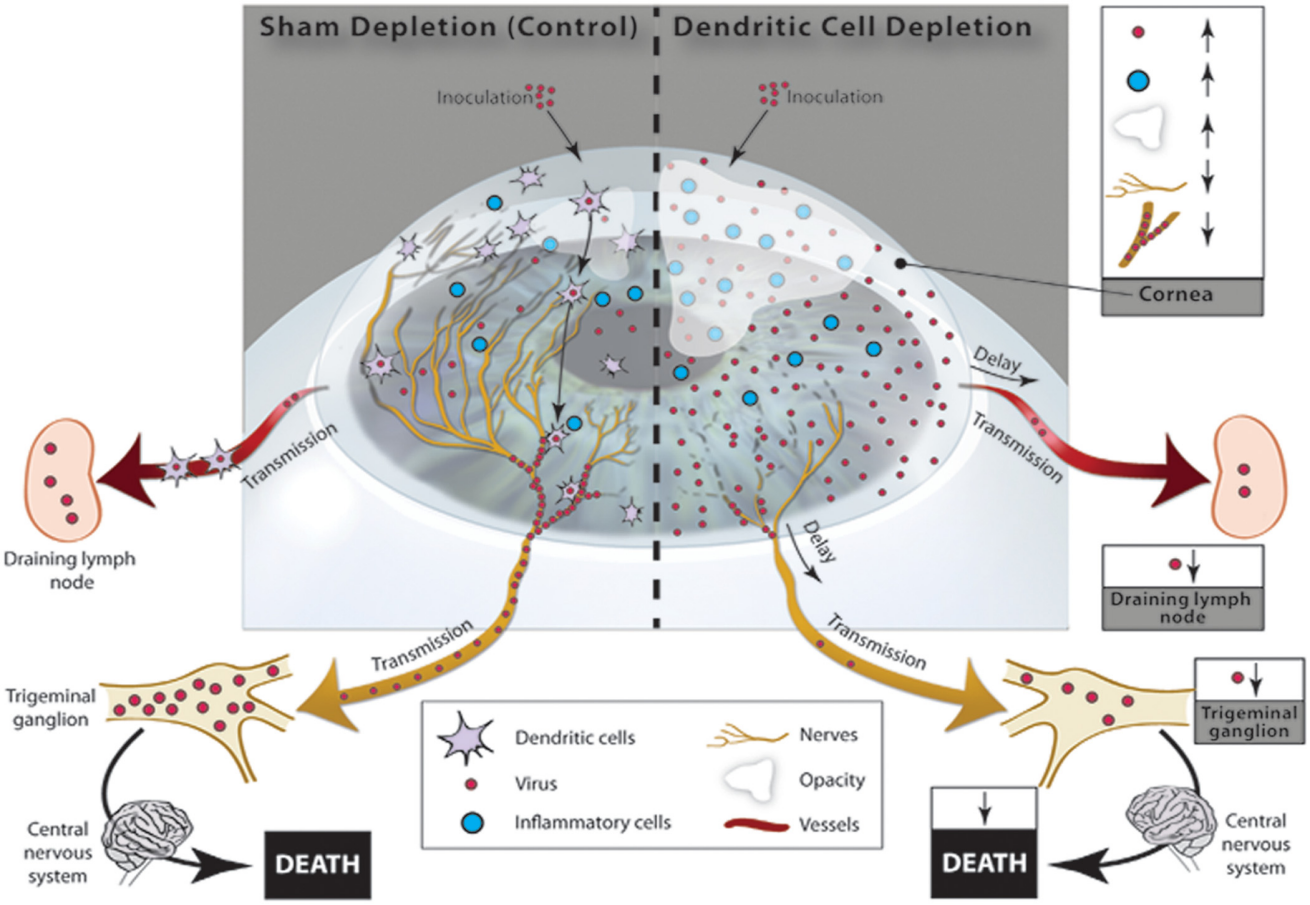
Corneal conventional dendritic cells play a pivotal role in the defense against HSV-1 virus, by reducing local corneal damage and preserving vision, at the cost of mediating systemic viral transmission. HSV-infected corneas demonstrate local viral replication, increased inflammatory cell infiltration, and corneal nerve damage, resulting in keratitis and corneal scarring. Infection of corneal nerves by HSV-1 may directly or indirectly be mediated through cDCs, resulting in transmission of the virus to the systemic compartment, including TGs, dLNs and the CNS, resulting in latency, or death. However, depletion of corneal cDCs results in increased viral proliferation, corneal inflammatory cells infiltration, nerve damage, and increased keratitis severity, scarring and blindness. Further, there are decreased corneal nerves infection, resulting in delayed and decreased systemic viral transmission to TGs and dLNs, and subsequent decrease in mortality. Collectively, corneal cDCs reduce local corneal damages and preserve vision after HSV infection, at the cost of mediating systemic viral systemic transmission resulting in death.

In summary, primary HSV-1 corneal epithelial infection results in clinical keratitis, viral replication, nerve damage, and increased leukocyte infiltration. Concurrently, peripheral corneal nerves are invaded by neurotropic viruses, such as HSV-1, and are transmitted to dLNs, bilateral TGs and even the CNS, resulting in death (Fig 7). However, cDCs, but not MΦs, play a key role in limiting corneal damage and preserving vision. While cDC depletion results in increased keratitis severity, limited neuronal viral invasion results in diminished systemic viral transmission and increased survival. Thus, corneal cDCs preserve vision at the cost of systemic viral transmission and increased latency, by shifting HSV from the local corneal compartment to the peripheral systemic compartments.

## Discussion

HSV-1 has been shown to directly infect the cornea primarily through the “front door” [3, 5, 39] before its transmission through corneal nerves to the TG, where it remains latent. The latent virus can then undergo re-activation, resulting in recurrent herpes stromal keratitis, which commonly results in corneal blindness. The reason as to why primary corneal infections do not result in scarring and vision loss has remained elusive to date, although corneal epithelial cells have been shown to express HSV entry receptors [3], rendering them susceptible to direct viral infection. Here we demonstrate how corneal cDC preserves vision in primary HSV-1 infection, at the cost of systemic transmission of the HSV-1 virus. Our data may explain the limited clinical findings on primary HSV-1 infections of the cornea. The fact that primary HSV infection of the cornea does clinically rarely result in corneal scarring in humans, supports our data in that presence and increase of cDCs in patient corneas may prevent scarring and blindness in primary acute keratitis, while resulting in latency.

The role of cDCs in primary herpes simplex keratitis, particularly in the initial phase of host-pathogen interaction, has remained elusive, partly due to the fact that the central cornea has historically been thought to be devoid of bone marrow-derived cells, including cDCs [40–41]. Increase in epithelial cDCs had been noted after HSV infection by others [12, 16, 42–44], but to date, this had been attributed to de novo migration into the cornea. The discovery of resident immature cDCs and MΦs in the central cornea [14, 29, 45], has led to a surge in interest in their role in corneal disease. The evidence that corneal cDCs undergo maturation as a result of corneal insult and inflammation, leads to the assertion that MHC-II^+^ LCs, previously described in herpes simplex keratitis, are not only due to de novo migration of these cells as previously thought, but also, to a large degree, due to maturation of resident cDCs. Consequently, the cascade of events during the initial HSV infection and the differential role of cDCs and MΦs were studied here.

The roles of cDCs in herpes simplex keratitis have recently been studied by some investigators. Ghiasi et al. mainly focused on the role of cDCs on HSV latency and HSV vaccination [15, 46]; or the role of CD11c in controlling the HSV virus replication [27]. Further, Hendricks et al. studied the role of cDCs in mediating infiltrating cells in the cornea, in particular NK cells [17]; or the role in the CD4^+^ T cell expansion [47] after HSV-1 ocular infection. But the detailed roles of corneal cDCs in disease development and outcome, especially in the interaction with nerves and viral dissemination, as well as the differential roles of corneal DCs vs. MΦs, in primary acute HSV-1 ocular infection remain unknown. Our study herein fills this gap.

Although several in vitro studies have demonstrated that immature and mature cDCs can be infected by HSV [48–51], the specific role of cDCs in the pathophysiology of HSV infection has been controversial. It has been shown that cDCs can migrate to local infection sites, are able to present HSV antigen to T cells and trigger CTL functions in vivo [52–55], and thus participate in resistance against HSV [56–59]. However, HSV down-regulates T cell responses by interrupting antigen processing [60], in part through down-regulation of co-stimulatory molecules and MHC-I [50, 61–64]. Yet other studies have shown that HSV-infected cDCs secrete low levels of interleukin (IL)-12, suggestive of a low maturation stage, but that infected cDCs can trigger bystander cDC maturation and cross-presentation of HSV antigens [61]. Here, we show that the cDC density increases *in vivo* within days of viral inoculation, and more importantly, in contrast to most previous in *vitro* studies, they do express MHC-II, indicating cDC maturation. Interestingly, this increase in central corneal cDC density was earlier and more profound than increase in MΦ density.

Systemic cDC-depletion with DT was surprisingly ineffective in depleting corneal cDCs and could be explained with the avascularity of the cornea. Thus, our modified s.c. DT application, similar to CL application previously described for MΦ depletion [65–66], was superior as compared to systemic depletion [17]. Moreover, recently Hendricks et al. reported a similar s.c. depletion model [47]. The rapid repopulation of corneal cDCs shown within 3 days despite lack of corneal vascularization, suggests that specific recruitment pathways are at play within the cornea. Our studies further demonstrate that cDC-depletion prior and during HSV inoculation results in rapid and severe clinical keratitis and scarring, as compared to controls. To determine the underlying pathophysiological mechanisms, we show that increased scarring of cDC-depleted corneas is associated with significant increase in corneal viral titers and viral mRNA, as well as increased stromal viral invasion. Kassim et al [67] have previously reported that systemic depletion of cDCs at a single time point of 12h before infection, resulted in increased viral load, systemic dissemination, and death. However, as these studies were performed in a non-ocular model of footpad injection (not a natural route for HSV entry), the relevance to a clinically relevant model of HSV infection remained unclear. Moreover, as the depletion was performed systemically and only at a single time point, the effect of local cDCs, as shown in our manuscript, could not be demonstrated, as cDCs are depleted in non-ocular tissues (including the brain) as well, and also repopulate within a few days.

We demonstrate severe corneal damages in cDC-depleted corneas, and increased inflammation. Theoretically, depletion of cDCs or cell death may cause inflammation, and subsequently limit HSV-1 infection. However, our study had an important control group, in which macrophages were depleted, causing cell death as well. This group, however, did not show an effect on either HSV-1 infection or on corneal inflammation, despite macrophage death. Thus, we believe that the changes we observe with cDC depletion are specific to cDCs and not due to cell death-related inflammation. The use of PSGL-1 KO mice, in which there is limited recruitment of immune and inflammatory cells through the blood, including myeloid-derived suppressor cells, shows an even higher level of nerve damage, suggesting that it is in fact the virus and not inflammation that results in nerve damage. We used β-III tubulin as the marker for nerve loss, as we would like to show whether the distal ends of the axons are damaged locally in the cornea or not. Our data shows that corneal nerve damage is not strain specific, and not related to scarification. Collectively we demonstrate that cDC depletion results in increased viral proliferation and subsequent corneal damage and that the presence of corneal cDCs preserves vision through limiting local damage.

While depletion of MΦs alone has no effect on keratitis and corneal damage, cDC/MΦ double-depletion demonstrates increased damage and keratitis as compared to cDC depletion alone, suggesting that MΦs may play a second line of defense in the cornea. These results differ from a previous report by Cheng et al [9], in which depletion of MΦs resulted in decreased viral titer at 8 days post infection, a time point at which HSV-1 is typically cleared from the cornea. Moreover, their methodology to deplete MΦs at 2 and 4 days before (but not after) infection, would have resulted in corneal repopulation of MΦs within 2–3 days as we have shown here. Interestingly, on days 2 and 4 after infection, a time point where MΦs may remain depleted or partially depleted, they also showed no differences in outcomes with MΦs depletion as compared to the control group, consistent with our results that show a lack of effect through MΦs depletion at the time at which viral titers in the cornea peak.

The decreased viral proliferation in the presence of corneal cDCs could, in part, be a result of enhanced NK cell activity [17]. Although Frank et al. also reported that systemic (not local) cDC depletion resulted in increased vital titers in tears, our study goes well beyond demonstration of increased viral titers after cDC depletion and provides novel roles for local cDC in viral diseases, including changes of clinical severity, modulation of the immune response, nerve damage, changes in cDC density and phenotype, the direct role of HSV on nerve destruction, the differential role of cDC and macrophages in acute viral disease in the peripheral tissue, and subsequent changes in systemic compartments. Most importantly, we demonstrated that local cDCs may mediate systemic viral transmission, which to our knowledge, has not been demonstrated in any viral disease.

A previous study by Hendricks and colleagues has shown that depletion of cDCs in one eye through UV-B irradiation, before bilateral HSV-1 infection, reduces the incidence and severity of late stromal disease in infected cDC-depleted corneas through induction of a T cell response [16]. However, while it has been postulated that HSV cannot replicate in infected cDCs [51, 68], other reports have demonstrated its replication in mature cDCs and the ability to transfer HSV in a cell—cell contact-dependent manner [69]. Given the intimate proximity of cDCs and corneal nerves, and the recently reported potential interaction of the immune and nervous system in the cornea in patients with infectious keratitis [70], we next tested the hypothesis that cDCs may be involved in viral transmission to corneal nerves. Our studies demonstrate that cDC-depletion results not only in increased corneal nerve damage, but also in significantly decreased and delayed viral infection of corneal nerves, resulting in limited systemic viral transmission. The decrease in systemic transmission results in accumulation of the virus and increased local corneal viral titer levels.

One of the important findings of our study is that decreased viral transmission to the TG with cDC depletion, results in decreased mortality from herpetic encephalitis [71]. Previously, Mott et al [46] have shown that cDC depletion in vaccinated mice resulted in chronic enhancement of HSV-1 latency. However, chronic latency and acute mortality may not be directly comparable, as many factors can affect latency, e.g. CD8+ T cell level. Further, they performed intraperitoneal injections of diphtheria toxin, which results in systemic depletion of cDCs (including the brain), and this may consequently alter the systemic immune responses leading to the changes of latency. In contrast, we directly demonstrate the locally decreased infectivity of corneal nerves upon cDC depletion, as well as subsequent decrease in trigeminal ganglion levels. Moreover, while we show, for the first time, that HSV-1 virus can transmit from the cornea to the dLNs, it is diminished with corneal cDC-depletion. In contrast, depletion of MΦs has no effect on systemic viral transmission. Thus, our data demonstrate that corneal cDCs preserve vision through limiting local corneal damage, at the cost of enhanced systemic viral transmission to peripheral compartments, resulting in latency [46] or death. These findings may apply to other neurovirulent viruses or other organs, potentially leading to new approaches to promote defense against viruses in the future. Future studies will determine if the enhanced viral transmission to the systemic compartment is due to direct cell (cDCs)-cell (nerves) dependent fashion, or through changes in the cytokine milieu in the cornea (cytokine storm). The detailed molecular mechanisms need to be investigated to demonstrate how cDCs specifically control the severity of infection in the cornea and mediate systemic transmission, Nevertheless, our data clearly demonstrates a direct or indirect role of cDCs in the spread of HSV virus from the cornea to the systemic compartment, thus contributing to decrease of viral load, providing a conceptual advance in this field.

Interestingly, and unexpectedly, our data shows bilateral viral transmission to the TG after unilateral ocular HSV infection, albeit with a 2-day delay. The contralateral corneas neither demonstrated clinical keratitis, nor the presence of HSV mRNA, suggesting transmission through the CNS. While previous studies have demonstrated bilateral abnormalities in sensory roots after unilateral varicella zoster virus infection [72], transmission of HSV to the contralateral TG had not been reported before.

To evade the host immune response, HSV induces apoptosis and elimination of attacking cDCs. In combination with a delayed activation of T cells by HSV-infected suppressed cDCs, these mechanisms may enable HSV to replicate for a longer time before effective defense strategies of the host are induced. While the HSV has developed several strategies to evade the host immune system, the host ocular immune response has in turn developed strategies to preserve vision, by shifting the virus from the local ocular compartment to the systemic compartment.

## Supporting Information

S1 Fig Local corneal conventional dendritic cell depletion by subconjunctival injection of diphtheria toxin is more effective than systemic depletion. **A:** Single subconjunctival (s.c.) injection of DT on day -2, resulted in almost completed corneal cDC depletion on day 0 p.i., as compared to 40% depletion by systemic intraperitoneal (i.p.) injection. Repeated s.c. injections, every 2 days, resulted in continuous corneal cDC depletion by more than 95% compared to only 50% depletion by i.p. injections on days 0, 3, 6 p.i. **B:** Single s.c. injection of clodronate liposomes (CL) on day -2 resulted in almost complete MΦ depletion on day 0 p.i., as compared to less than 40% depletion by i.p. injection. Repeated s.c. injections every 2 days resulted in continuous depletion of MΦ by more than 95% compared to 40% depletion with i.p. injections on days 0, 3, 6 p.i. *p<0.0001 compared with s.c. (Student’s t-test). **C:** Corneal cDCs and MΦs are successfully depleted simultaneously. After a single s.c. injection of both DT and CL on day -2 p.i., cDCs and MΦs were almost completely depleted on day 0 p.i. On day 3 p.i, the corneas started to repopulate with cDCs (65%) and MΦs (80%), and increasing to more than 82% and 87% respectively on day 6 p.i. With repeated injections of DT and CL every 2 days, continuous depletion of both cDCs and MΦs was achieved by more than 90% on days 0, 3, 6 p.i. **D, E, F:** s.c. DT injection did not deplete the cDCs of dLN (submandibular lymph nodes). **D,** The representative micrograph of cDCs in dLN after s.c. saline injection (sham control). **E,** The representative micrograph of cDCs in dLN after s.c. DT injection. **F,** There was no significant difference in cDC density in dLN between DT and control saline injections. *P*>0.05 (Student’s t-test). Scale bar: 200 μm. Data are shown as mean ± SD.
(TIF)

S2 Fig Dynamic changes of corneal inflammatory cell infiltration after HSV-1 infection with corneal conventional dendritic cell and macrophage depletion. Depletion of conventional dendritic cells (cDCs), but not macrophages results in continuous increase of corneal inflammatory cells as compared to sham-depleted and macrophage-depleted corneas. *p<0.05, **p<0.01 compared with sham-depleted. ^**#**^ *p*<0.05 compared with cDC(-)(ANOVA). Data are shown as mean ± SD.
(TIF)

S3 Fig Corneal nerve damage in detail after HSV-1 infection with corneal conventional dendritic cell and macrophage depletion. **A:** Dynamic changes of corneal nerve damage. Depletion of corneal conventional dendritic cells, but not macrophages results in continuous and more severe damage of corneal nerves as compared to sham-depleted and macrophage-depleted corneas. *p<0.05, **p<0.01 compared with sham-depleted; ^**#**^ *p*<0.05 compared with cDC(-) (ANOVA). **B, C, D, E:** Corneal nerve damage are not related to scarification, and not strain specific. **B,** The representative micrograph of corneal nerves in a normal cornea. **C,** The representative micrograph of corneal nerves with HSV-1 infection without scarification. **D,** The representative micrograph of corneal nerves with HSV-1 KOS strain infection. **E,** Corneal nerves were significantly reduced in the mice with HSV-1 infection without scarification or with HSV-1 KOS strain infection as compared to the normal mice. *p<0.05, **p<0.01 compared with normal (Student’s t-test). Scale bar: 100μm. **F:** Dendritic cells demonstrate close anatomical proximity with corneal nerves. Scale bar: 200 μm. Data are shown as mean ± SD.
(TIF)

S4 Fig HSV-1 viral mRNA was detected in contralateral trigeminal ganglia after ipsilateral corneal infection, without evidence for contralateral keratitis or viral corneal mRNA. **A**: Representative clinical corneal photographs demonstrating no keratitis in the contralateral corneas as compared to infected corneas. **B:** No HSV-1 mRNA was detected in contralateral corneas, but was present in ipsilateral corneas. **C:** Viral titer levels were detected in contralateral and ipsilateral trigeminal ganglia.
(TIF)

S1 Movie The movie demonstrates HSV-1 virus (green) located within the dendritic cells (red), suggesting infection of conventional dendritic cells in vivo by HSV-1. (AVI)
