# Supplementary figures and images for: A Dual Role for Corneal Dendritic Cells in Herpes Simplex Keratitis: Local Suppression of Corneal Damage and Promotion of Systemic Viral Dissemination
Source: PLoS One. 2015 Sep 2;10(9):e0137123. doi: 10.1371/journal.pone.0137123 (PMC4557979; doi:10.1371/journal.pone.0137123)

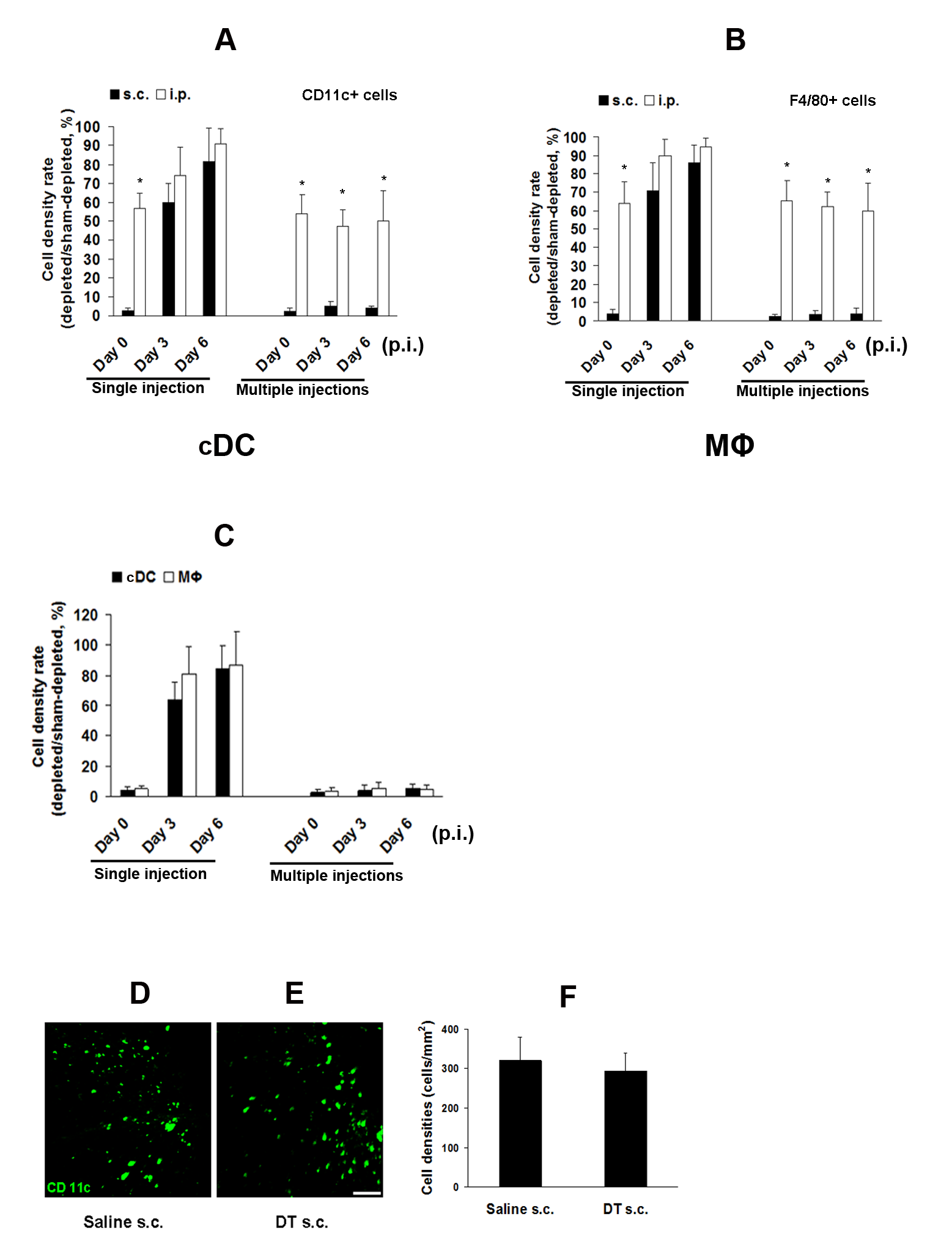

Supplement: S1 Fig — A: Single subconjunctival (s.c.) injection of DT on day -2, resulted in almost completed corneal cDC depletion on day 0 p.i., as compared to 40% depletion by systemic intraperitoneal (i.p.) injection. Repeated s.c. injections, every 2 days, resulted in continuous corneal cDC depletion by more than 95% compared to only 50% depletion by i.p. injections on days 0, 3, 6 p.i. B: Single s.c. injection of clodronate liposomes (CL) on day -2 resulted in almost complete MΦ depletion on day 0 p.i., as compared to less than 40% depletion by i.p. injection. Repeated s.c. injections every 2 days resulted in continuous depletion of MΦ by more than 95% compared to 40% depletion with i.p. injections on days 0, 3, 6 p.i. *p<0.0001 compared with s.c. (Student’s t-test). C: Corneal cDCs and MΦs are successfully depleted simultaneously. After a single s.c. injection of both DT and CL on day -2 p.i., cDCs and MΦs were almost completely depleted on day 0 p.i. On day 3 p.i, the corneas started to repopulate with cDCs (65%) and MΦs (80%), and increasing to more than 82% and 87% respectively on day 6 p.i. With repeated injections of DT and CL every 2 days, continuous depletion of both cDCs and MΦs was achieved by more than 90% on days 0, 3, 6 p.i. D, E, F: s.c. DT injection did not deplete the cDCs of dLN (submandibular lymph nodes). D, The representative micrograph of cDCs in dLN after s.c. saline injection (sham control). E, The representative micrograph of cDCs in dLN after s.c. DT injection. F, There was no significant difference in cDC density in dLN between DT and control saline injections. P>0.05 (Student’s t-test). Scale bar: 200 μm. Data are shown as mean ± SD. (TIF) [file pone.0137123.s001.tif]

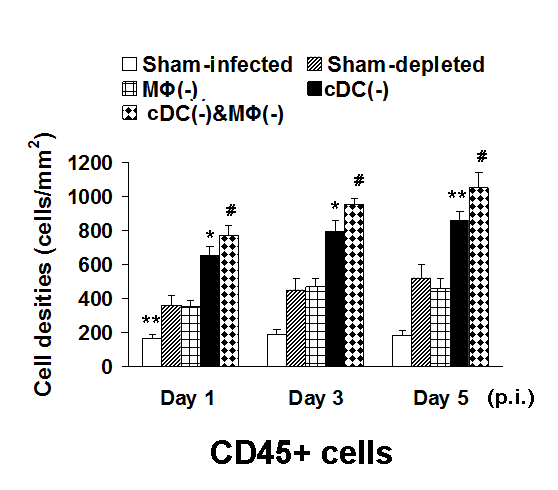

Supplement: S2 Fig — Depletion of conventional dendritic cells (cDCs), but not macrophages results in continuous increase of corneal inflammatory cells as compared to sham-depleted and macrophage-depleted corneas. *p<0.05, **p<0.01 compared with sham-depleted. # p<0.05 compared with cDC(-)(ANOVA). Data are shown as mean ± SD. (TIF) [file pone.0137123.s002.tif]

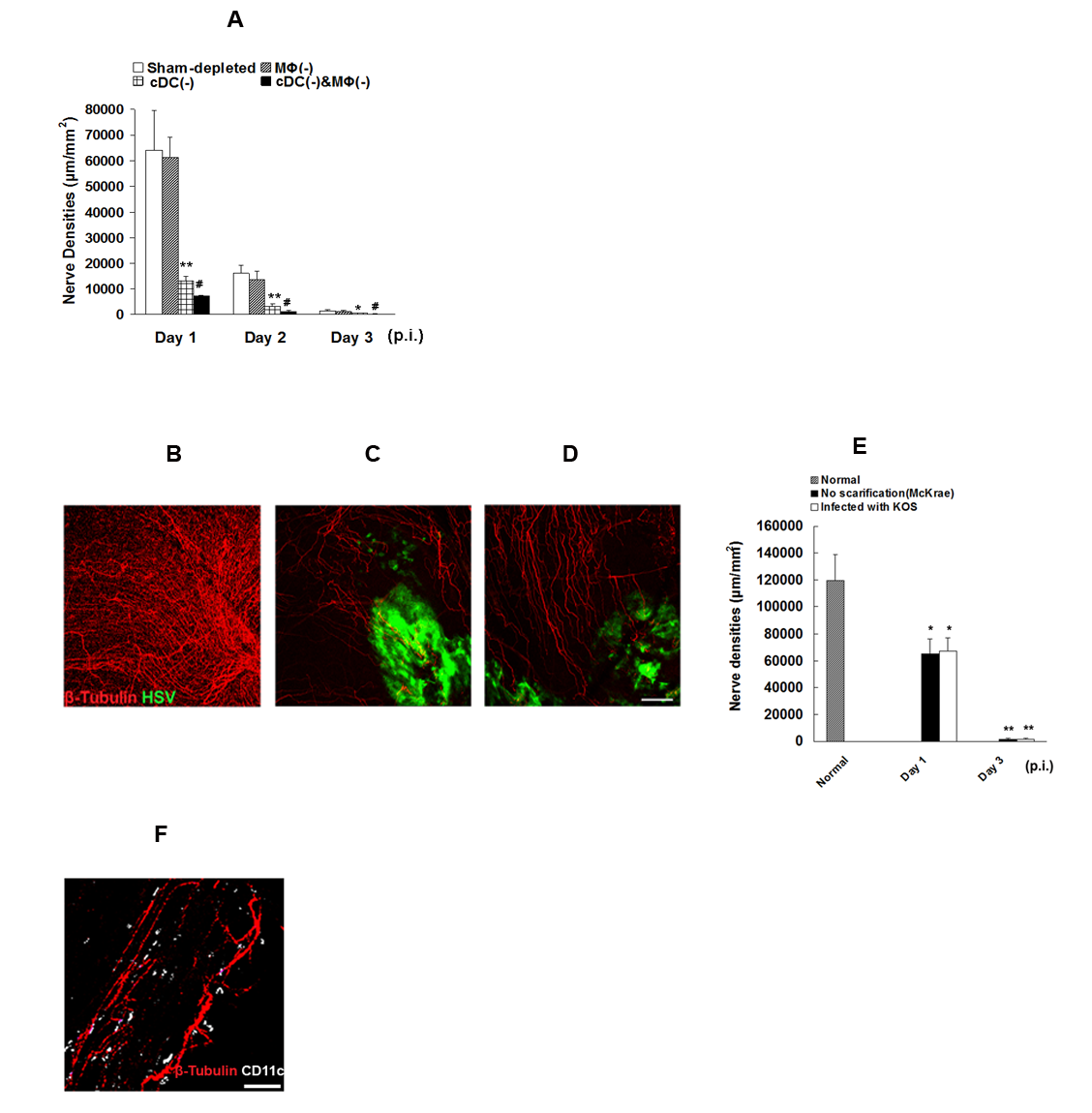

Supplement: S3 Fig — A: Dynamic changes of corneal nerve damage. Depletion of corneal conventional dendritic cells, but not macrophages results in continuous and more severe damage of corneal nerves as compared to sham-depleted and macrophage-depleted corneas. *p<0.05, **p<0.01 compared with sham-depleted; # p<0.05 compared with cDC(-) (ANOVA). B, C, D, E: Corneal nerve damage are not related to scarification, and not strain specific. B, The representative micrograph of corneal nerves in a normal cornea. C, The representative micrograph of corneal nerves with HSV-1 infection without scarification. D, The representative micrograph of corneal nerves with HSV-1 KOS strain infection. E, Corneal nerves were significantly reduced in the mice with HSV-1 infection without scarification or with HSV-1 KOS strain infection as compared to the normal mice. *p<0.05, **p<0.01 compared with normal (Student’s t-test). Scale bar: 100μm. F: Dendritic cells demonstrate close anatomical proximity with corneal nerves. Scale bar: 200 μm. Data are shown as mean ± SD. (TIF) [file pone.0137123.s003.tif]

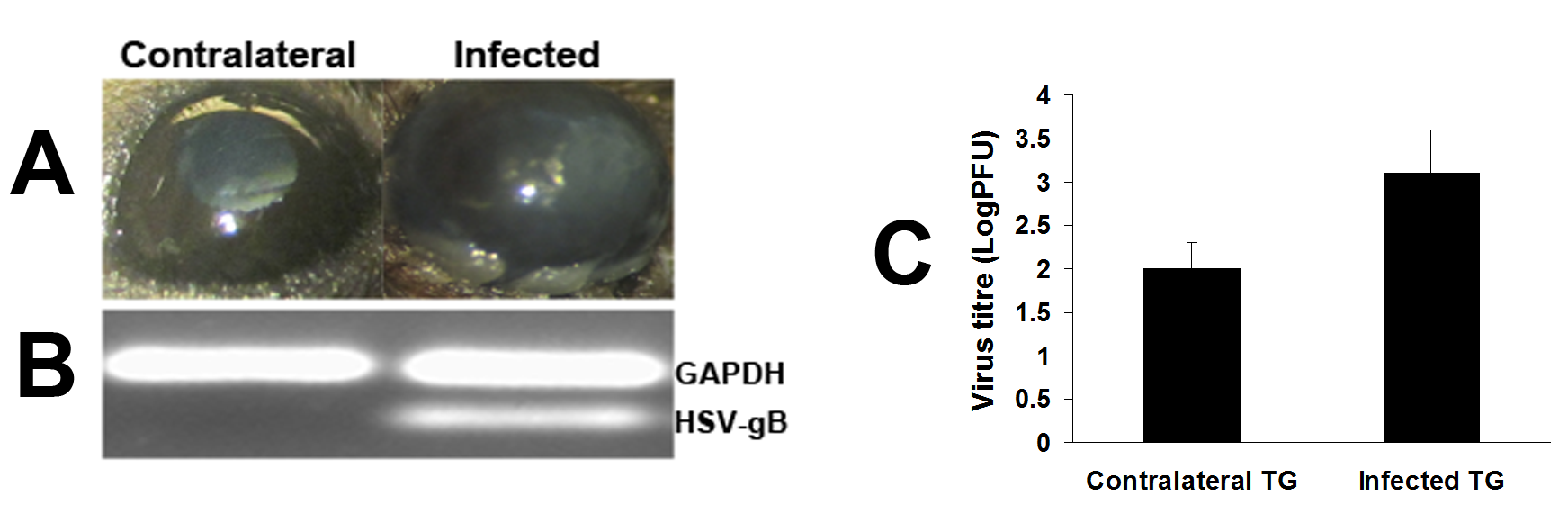

Supplement: S4 Fig — A: Representative clinical corneal photographs demonstrating no keratitis in the contralateral corneas as compared to infected corneas. B: No HSV-1 mRNA was detected in contralateral corneas, but was present in ipsilateral corneas. C: Viral titer levels were detected in contralateral and ipsilateral trigeminal ganglia. (TIF) [file pone.0137123.s004.tif]
